# Comparative Study of Carborane- and Phenyl-Modified Adenosine Derivatives as Ligands for the A2A and A3 Adenosine Receptors Based on a Rigid in Silico Docking and Radioligand Replacement Assay

**DOI:** 10.3390/molecules23081846

**Published:** 2018-07-25

**Authors:** Marian Vincenzi, Katarzyna Bednarska, Zbigniew J. Leśnikowski

**Affiliations:** 1 Laboratory of Molecular Virology and Biological Chemistry, Institute of Medical Biology of the Polish Academy of Sciences, 106 Lodowa St., 93-232 Lodz, Poland; marian.vincenzi@unina.it; 2 Laboratory of Experimental Immunology, Institute of Medical Biology, Polish Academy of Sciences, 106 Lodowa St., 93-232 Lodz, Poland; kbednarska@cbm.pan.pl

**Keywords:** in silico screening, adenosine, boron cluster, adenosine receptors, AR ligands

## Abstract

Adenosine receptors are involved in many physiological processes and pathological conditions and are therefore attractive therapeutic targets. To identify new types of effective ligands for these receptors, a library of adenosine derivatives bearing a boron cluster or phenyl group in the same position was designed. The ligands were screened in silico to determine their calculated affinities for the A2A and A3 adenosine receptors. An virtual screening protocol based on the PatchDock web server was developed. In the first screening phase, the effects of the functional group (organic or inorganic modulator) on the adenosine ligand affinity for the receptors were determined. Then, the lead compounds were identified for each receptor in the second virtual screening phase. Two pairs of the most promising ligands, compounds **3** and **4**, and two ligands with lower affinity scores (compounds **11** and **12,** one with a boron cluster and one with a phenyl group) were synthesized and tested in a radioligand replacement assay for affinity to the A2A and A3 receptors. A reasonable correlation of in silico and biological assay results was observed. In addition, the effects of a phenyl group and boron cluster, which is new adenosine modifiers, on the adenosine ligand binding were compared.

## 1. Introduction

Adenosine is a key endogenous molecule involved in the activation of the A1, A2A, A2B and A3 adenosine receptors (ARs), which belong to the P1 class of purinergic receptors. Each of these receptors promotes a different signaling pathways associated with specific, although with some overlap, effects. ARs are members of the G protein coupled receptors family, which also includes many well-known receptors, such as dopamine, adrenergic, histamine and serotonin receptors.

ARs in response to adenosine binding trigger essential signals into cells by activating one or more heterotrimeric G protein, located on the inner side of the cell membrane and subsequently influence multiple-effector systems (i.e., adenylate cyclase, ion channels, phospholipases). These receptors are important pharmacological and therapeutic targets [1].

**Figure 1:**
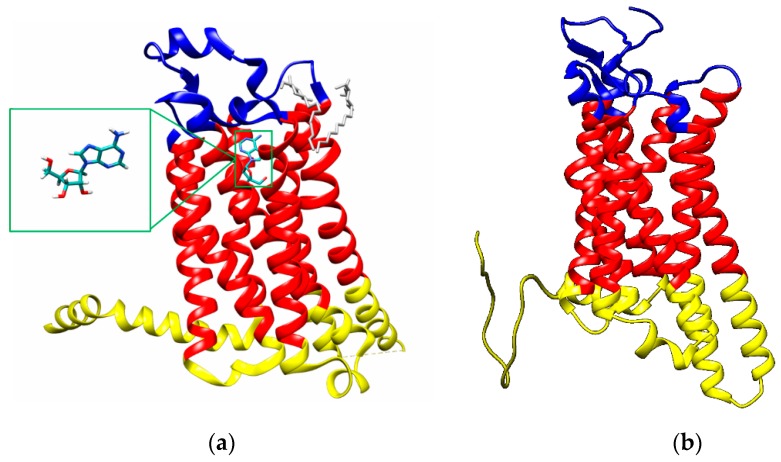
(**a**) X-ray structure of the thermostabilized human A2A receptor with bound adenosine (PDB code: 2YDO [28]; blue: extracellular region, red: transmembrane domain, yellow: cytoplasmic region); insert: adenosine; (**b**) A3 adenosine receptor structures obtained with LOMETS (cyan rectangle: A8.60-E318 region, blue: extracellular region, red: transmembrane domains, yellow: cytoplasmic region).

Similar to other G protein-coupled receptors, ARs consist of seven transmembrane helices that contain a ligand binding site. Each helix is composed of approximately 21 to 28 amino acids. The transmembrane helices are connected by three extracellular and three cytoplasmic loops with different numbers of amino acids. The N-terminus and C-terminus are located on the extracellular and cytoplasmic sides, respectively, of the cell membrane (Figure 1) [2]. For more than three decades, medicinal chemistry research has focused on developing potent and selective synthetic AR agonists and antagonists as agents potentially useful in the treatment of inflammation, the central nervous system (CNS) disorders and pulmonary or cardiovascular diseases. In addition, several allosteric modulators of AR subtypes have also been synthesized [3].

AR action can be modulated directly by ligands or indirectly by availability of extracellular adenosine through its metabolism or cellular uptake [3]. The A2A and A3 adenosine receptors are considered to be among the attractive therapeutic targets for the inflammatory disorders and cancer treatment [3]. In the development of potential drugs targeting ARs, many adenosine derivatives and non-nucleoside molecules have been synthesized and tested [4,5,6]. The presence of the hydrophobic pharmacophore is considered as one of the essential features of the ligands in terms of the binding activity and their A2A AR selectivity. One new avenue of research in this field is the development of nucleoside-boron cluster conjugates, including adenosine derivative conjugates.

Medicinal chemists are increasingly utilizing boron clusters (polyhedral boron cages) as a new generation of 3-dimensional, abiotic privileged scaffolds, modifiers and pharmacophores in bioactive molecule design [7,8,9,10]. Many boron cluster conjugates with biologically important low molecular weight compounds, including amino acids, lipids, carbohydrates, porphyrins, nucleic acid bases, nucleosides and DNA groove binders, have been synthesized [11,12,13,14]. In addition, biopolymers bearing one or more boron cages (carboranes), including carboranyl peptides and proteins, carboranyl oligophosphates, and nucleic acids (RNA and DNA oligonucleotides), have been prepared [13,15,16].

The low molecular weight biomolecules that have been conjugated to a boron cluster include many receptor ligands, such as estrogen, androgen, retinoic acid, dihydrofolate, etc. [8]. Boron clusters bearing ligands to ARs have also been described [17,18].

We previously reported the chemical synthesis of various nucleoside-boron cluster conjugates [15], including those formed from adenosine and evaluated their activity as blood platelet aggregation inhibitors [18], reactive oxygen species (ROS) inhibitors [19], antivirals [20] and anti-tumor agents [21]. Some of these compounds are also potential ligands to purinergic receptors [19,22,23,24]. Herein, a series of adenosine derivatives modified with either phenyl or boron cluster (carborane group) were evaluated to compare the effects of organic and inorganic modification on the ligand affinity for the A2A and A3 receptors, in silico. The approach to computational ligand-adenosine receptor rigid docking was applied to screened virtually adenosine conjugates bearing such diverse structures as the phenyl group and the boron cluster. Two pairs of ligands with the highest and lowest affinity scores were selected based on in silico screening results, then were synthesized and tested in vitro in the radioligand replacement assay. Finally, the results of in silico and in vitro study were compared.

## 2. Results and Discussion

Due to the limitations of the available docking algorithms and the unique properties of boron clusters that prevent them from being defined in the same way as organic moieties [10,25], higher errors are obtained in in silico studies of boron clusters than in those of purely organic structures. Herein, a simple and versatile computational approach based on PatchDock web server was used providing preliminary information on ligand receptor interaction. The results of the in silico screening were verified by the synthesis of the real compound library followed by in vitro screening of the obtained ligands. This work provides insight into the effects of boron clusters on the adenosine affinity for the A2A and A3 receptors and the relationship between in silico and experimental results. Here, proof of concept of the presented in silico assay and its comparison with radioligand replacement test is described.

It is generally accepted that in silico target profiling methods for selecting lead compounds are efficient alternatives to expensive, time-consuming high-throughput in vitro target profiling of compound libraries. This type of approach was recently used to successfully identify molecules that selectively bind to the A1, A2A, A2B and A3 receptors [26]. The selected molecules were further evaluated in vitro under the same conditions. This work utilizes a similar approach though with other focuses. The main difference between the previous study and the study presented herein is that instead of screening large, diverse compound libraries, a small specialized library of adenosine derivatives was utilized, and the effects of specific, known phenyl group modification were compared to those of the corresponding inorganic modifications with boron cluster cage. This task oriented approach allowed to evaluate the effects of boron cluster modification on adenosine ligand properties [7,10,27] and to propose a practical protocol for comparative study in silico.

### 2.1. Protein Structure Selection and Modeling

#### 2.1.1. A2A Adenosine Receptor

The choice of the protein structure is critical in virtual screening studies. In this work, we studied two different adenosine receptors i.e., A2AR and A3R. For the A2A adenosine receptor model, the crystal structure of the thermostabilized human A2A receptor with bound adenosine located in the binding pocket was selected (Protein Data Bank (PDB) code 2YDO, Figure 1a) [28]. The A2A receptor structure was extracted from the X-ray structure using the UCSF Chimera visualization system [29]. The receptor structure is fixed in the active state.

#### 2.1.2. A3 Adenosine Receptor

Because the A3 adenosine receptor structure has not yet been deposited in the PDB, protein homology modeling [30,31,32] was performed using a procedure previously reported in the literature [30] and LOMETS (Local Meta-Threading-Server) [33], one of the many programs available for this purpose. The amino acid sequence of A3 AR was obtained from the UniProtKB/Swiss-Prot database (P0DMS8).

Using LOMETS, PDB 5IU4 structure of the complete A2 AR [34] was selected and verified as the best fit for the designing of A3 AR model with the highest score. According the validation of the 5IU4 model as the template for A3 AR, the conformation of 5IU4 structure gives similar fashion of adenosine binding like in 2YDO. The A3 adenosine receptor structure obtained by Lomets modeling is reported in Figure 1b.

### 2.2. Docking Method Validation and Optimization

#### 2.2.1. A2A Adenosine Receptor

**Figure 2:**
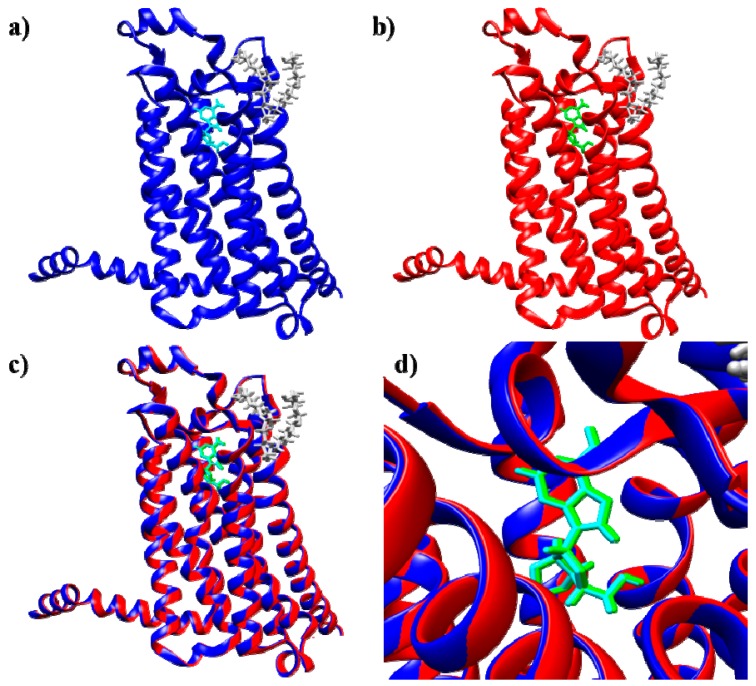
(**a**) X-ray structure of the thermostabilized human A2A receptor with bound adenosine (PDB code: 2YDO) [28]; (**b**) best docked pose for adenosine obtained with SwissDock; (**c**) superimposition of the reference complex (PDB code: 2YDO) and the best docked adenosine pose; (**d**) magnification of the binding pocket viewed from the top of the extracellular region (blue and cyan: A2A receptor and adenosine, respectively, in the X-ray reference structure; red and green: protein and adenosine, respectively, in the best docked structure; yellow: 1-*S*-octyl-β-d-thioglucoside molecules (thioglucoside), crystallization helper molecules).

To validate the SwissDock docking methodology, a study was performed using adenosine as the reference ligand (Figure 1a), and the best docked structure (Figure 2b) was compared to the X-ray structure of the A2A receptor/adenosine complex (PDB code: 2YDO [28], Figure 1a and Figure 2a. The superimposition of the two structures showed that the position and orientation of adenosine in the best docked pose are identical to those in the crystal 2YDO structure (Figure 2c,d), showing that the SwissDock web server method [35] is valid for this ligand-receptor system. In this context it is important to consider that SwissDock web server was chosen because it is a program that give the possibility to consider the flexibility of the side chains of residues into input protein, thus giving a chance to better describe the protein-ligand interaction.

The docking mode is “blind”, i.e., without any specific box of analysis which results in consideration of the entire surface of the protein by the docking program. In our case the reference structure for A2A receptor (PDB code: 2YDO) is characterized by the presence of crystallization helper molecules that normally are not present and therefore were removed in docking experiments. One may judiciously expect that helper molecules would not influence the binding pose at the extracellular pocket attained by the docking, however to make sure that this custom change does not influence docking results docking experiments with and without the helper molecule were performed. In the selected A2A structure (PDB code: 2YDO), two sets of SwissDock control docking simulations, one with and one without the crystallization helper molecules [28], were performed with adenosine as the ligand (Figure S1a,b). As expected, the superimposition of the two best A2A receptor/adenosine structures obtained from these simulations revealed that the presence of the crystallization helper molecules does not influence the protein-ligand interactions (Figures S1 and S3).

#### 2.2.2. A3 Adenosine Receptor

A3 AR structure obtained by homology modeling with LOMETS [33] was validated with the use of two different tools, VERIFY3D [36] and RAMPAGE (Ramachandran plot analysis) [37]. The VERIFY3D program compares the overall structure to the amino acid sequence using a 3D profile computed from the atomic coordinates of the given structure and it has been used to determine the accuracy of the A3R model obtained using LOMETS.

The VERIFY3D analysis revealed that the structure of A3 AR has high percentage of residues with an average 3D-1D score of <0.2, indicating that our 3D model is compatible with its sequence.

The RAMPAGE program provides tools for analyzing Ramachandran plots to assess the stereochemical quality of proteins and the distribution of residues between the different regions (“favored”, “allowed” or “outlier”). It should be noted that in our study the number of residues classified as the favored regions is prevailing. Overall, the results obtained both by VERIFY3D and RAMPAGE homology modeling tools, confirmed the validity of our A3R model.

#### 2.2.3. Ligand Modeling

**Figure 3:**
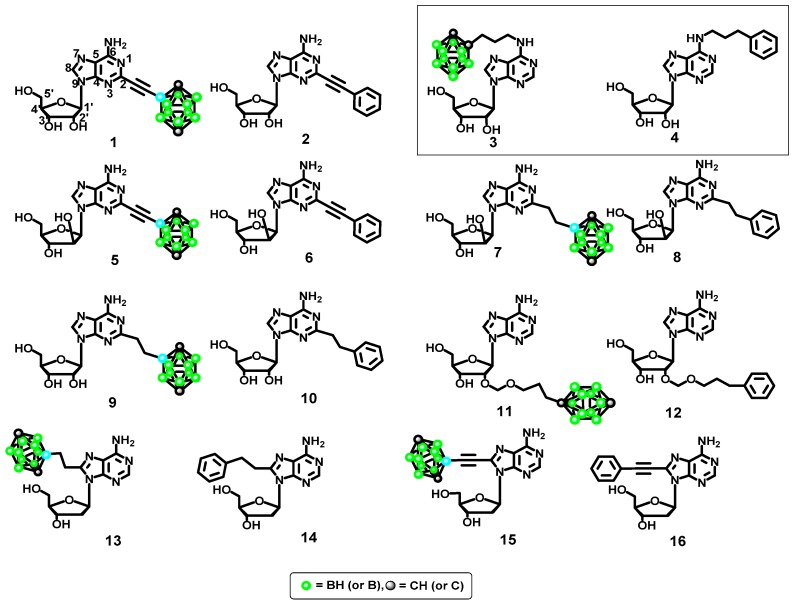
Modified adenosine derivatives designed as potential ligands for the A2A and A3 adenosine receptors screened in silico (dodecahedral structures correspond to the 1,12-dicarba-*closo*-dodecaboran-1-yl substituent, C_2_B_10_H_11_).

Modeling of the set of ligands was the next stage of our docking study (Figure 3). Figure 3 shows the series of molecules **1**–**16** designed to test the effects of adenosine structural modifications on the interactions between the ligand and the A2A and A3 purinergic receptors. Four specific modifications were evaluated: (1) type of lipophilic group (phenyl ring vs. boron cluster); (2) sugar configuration (β-d-ribofuranose vs. β-d-arabinofuranose); (3) position of the adenosine modification (2, 8 or 2′) and (4) spacer flexibility (ethynyl vs. ethyl linker).

Here, the boron clusters present an interesting challenge. Various docking approaches for molecules with boron clusters have been reported in the literature. One method is to substitute a carborane cage (C_2_B_10_H_12_) with one of its common bioisosteres, such as aryl, cycloalkyl and adamantyl groups [38,39,40]. The advantage of this method is that all the atom types are well described in the available docking programs; however, the steric properties of the boron cluster are not properly described. Therefore, others prefer to use protocols that can be directly applied to boron cluster structures.

It should be noted that most of available docking software such as AutoDock, FlexX, Glide, and Surflex do not have built-in parameters for hexacoordinated boron atoms, meaning calculations of molecules containing these atoms cannot be performed [40]. The most widely used approach to solve this problem is to change the boron atom type to the C.3 atom type [41,42,43,44]. In accordance with this approach, boron clusters are artificially treated as clusters of only carbon atoms. Using this protocol, some new information about the effect of the boron cluster structure on the protein-ligand interactions can be obtained. However, the effects of specific boron cluster properties [45], such as 3D aromaticity, hydridic character of the B-H hydrogens, dihydrogen bond formation or sigma-hole bonding, on ligand and receptors interaction cannot be determined in this simplified model. Therefore, herein the shape complementarity approach was applied to screen boron-bearing adenosines as the ligands for AR and the PatchDock software [46], as a tool for rigid ligand docking was used. The PatchDock provided a way to get preliminary information on ligand-protein interaction energies and contact surfaces without the change of the boron cage atom types, though although without consideration of the properties of boron cluster listed above.

PatchDock is a geometry-based molecular docking algorithm based on the object recognition and the image segmentation procedures used in Computer Vision that searches for docking configurations with good molecular shape complementarity. The PatchDock program assigns each molecule a geometric shape complementarity score (geometric score) that takes into account the interface area and desolvation energy associated with the protein-ligand interactions. Different candidate complexes are determined and ranked by a score that depends on the shape complementarity. The advantage of this docking program is the molecule input format. Whereas many methods require a “.mol2” input file format which cannot recognize the carborane boron and carbon atom types, PatchDock requires PDB input files. In this format the boron cluster structures can be more accurately described and thus the effect of this moiety on the space fitting of the entire molecule can be more precisely defined. Hence, all the docking studies in this work were performed using the PatchDock server. First, this program was validated using the same protocol as that used for the SwissDock web server. The obtained results revealed that as in the previous case, the method is reliable and provides correct information about the position of the molecule in the binding pocket of the A2A receptor (Figure S3). The slight shift of the adenosine position between the crystal structure and docking pose can be explained by the fact that PatchDock server does not consider flexibility of the residues side chains. However, this approximation did not affect the final result of the classification of our ligands.

#### 2.2.4. Reference Ligands

**Figure 4:**
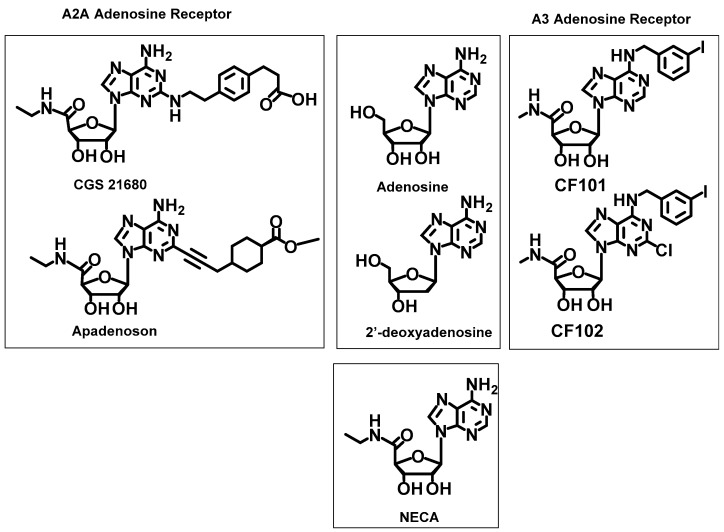
Reference ligands used to validate the PatchDock docking methods.

For the docking simulations, selected molecules that were reported to be efficient ligands for the A2A and A3 receptors were used as references (Figure 4) The selective agonists of A2AR, i.e., apadenoson (also known as ATL-146e, K_i_ = 0.68 ± 0.1 nM [47], phase IIb/III clinical trials [48]) and CGS 21680 (K_i_ = 17.3 ± 5.1 nM) [47] were chosen as the reference ligands for docking study. Additionally, the antagonist A2A, SCH 58261 (K_i_ = 1.3 nM) [49], was considered in calculations.

The selective agonists CF101 (known generically as IB-MECA, Ki = 1.8 nM, phase IIb/III clinical trials) [48] and CF102 (known generically as CI-IB-MECA, Ki = 1.4 nM, phase IIb/III clinical trials) [48] were chosen for the A3 receptor. Furthermore, NECA was also used for comparison (Figure 4) because it has a high affinity for adenosine receptors, although it does not exhibit receptor selectivity. Moreover, non-selective ligands, adenosine and 2′-deoxyadenosine, were used as the references.

### 2.3. Docking Results

As described in the “Docking method validation and optimization” section, the SwissDock and PatchDock docking methods were shown to give similar results. Indeed, the best docked adenosine pose was nearly identical in position and orientation to the reference crystal structure (PDB code: 2YDO) [28] (Figure 2d and Figure S2d). In the first screening phase, performed by PatchDock calculations, the effects of the functional group (organic or inorganic modulator) on the adenosine ligand affinity for the receptors were compared.

#### 2.3.1. Phenyl Ring vs. Boron Cluster

**Figure 5:**
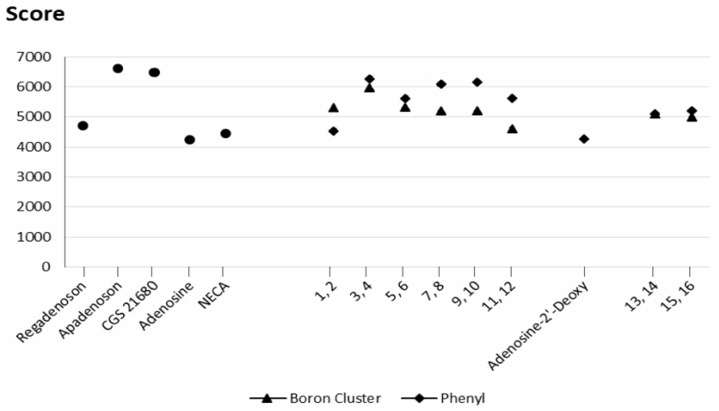
Geometric scores for the ligand shape complementarity to the A2A receptor. ● reference molecules (apadenoson, CGS 21680, adenosine, 2′-deoxyadenosine, NECA and SCH 58261; ▲ molecules bearing a boron cluster (**1**, **3**, **5**, **7**, **9**, **11**, **13**, **15**); ◆ molecules with a phenyl group (**2**, **4**, **6**, **8**, **10**, **12**, **14**, **16**).

Figure 5 shows the geometric scores for the docked molecules **1**–**16** in the A2A receptor (for the exact numerical values, see the Supplementary Information, Table S1). The calculations revealed that specific ligands were described by the score above 5000–5200 (apadenoson, CGS 21680 and SCH 58261), and non-specific ones by scores below this threshold (adenosine, 2′-deoxyadenosine, and NECA, Figure 5). A comparison of geometric scores for adenosine derivatives revealed that the molecules with phenyl groups (Figure 5) have similar (compounds **4**, **6**, **14**, and **16**) or higher (compounds **8**, **10**, and **12**) geometric scores than the corresponding molecules bearing boron clusters (compounds **1**, **3**, **5**, **7**, **9**, **11**, **13** and **15**). Interestingly, compound **1**, which has a boron cluster at position 2, has a higher geometric score than the corresponding molecule with a phenyl group (compound **2**) (Figure 5).

For the compounds within the pairs **3**–**4** and **5**–**6**, the absolute differences in the affinity scores are small, but they vary slightly between the pairs. For the pairs **13**–**14** and **15**–**16**, the scores are nearly identical. However, the presence of a large, rigid group such as a boron cluster can hinder deep penetration of the ligand into the binding pocket of the receptor (compounds **7**, **9**, and **11**).

**Figure 6:**
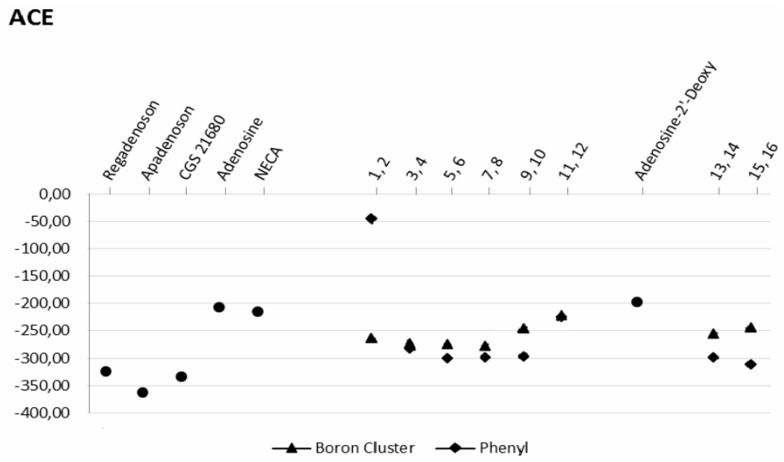
Desolvation energy (ACE) for the A2A protein-ligand interactions. ● reference molecules (apadenoson, CGS 21680, adenosine, 2′-deoxyadenosine, NECA and SCH 58261; ▲ molecules bearing a boron cluster (**1**, **3**, **5**, **7**, **9**, **11**, **13**, **15**); ◆ ligands with a phenyl group (**2**, **4**, **6**, **8**, **10**, **12**, **14**, **16**).

As shown in Figure 6, the atomic contact energies (ACE), defined as the desolvation free energies required to transfer atoms from water to a protein’s interior, of compounds **7** and **9** are consistent with this observation. It should be noted that the desolvation energy of compound **1** (boron cluster at position 2) is much lower than that of compound **2** (phenyl group at position 2) (Figure 6).

**Figure 7:**
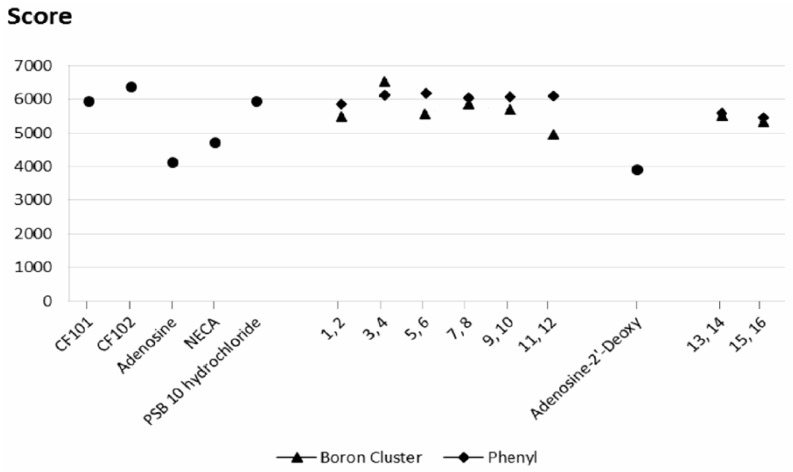
Geometric scores for the ligand shape complementarity to the A3 receptor. ● reference molecules (CF101, CF102, adenosine, 2′-deoxyadenosine, NECA and PSB 10 hydrochloride); ▲ molecules bearing a boron cluster (**1**, **3**, **5**, **7**, **9**, **11**, **13**, **15**); ◆ ligands with a phenyl group (**2**, **4**, **6**, **8**, **10**, **12**, **14**, **16**).

Similarly to results for A2AR, the score above 5000–5200 also described specific ligands for A3 receptor (CF101, CF102 and PSB 10 hydrochloride), and scores below this threshold non-specific ones (adenosine, 2′-deoxyadenosine and NECA, Figure 7). The affinity scores of compounds **1**–**16** for the A3 receptor are shown in Figure 7, and the exact values are listed in Table S2. In general, the affinity scores and desolvation energy profiles for binding to the A3 receptor are similar to those for binding to the A2A receptor (Figure 7 and Figure S4), although the differences between specific modifications are less pronounced. Furthermore, the A3 receptor appears to be less discriminative for the phenyl group than for the boron cluster. Similar to the A2A receptor results, both the phenyl and boron cluster modifications to the exo-amine group in position 6 are the most favorable.

These results indicated that the use of phenyl groups or boron clusters in the design of selective ligands for these two receptors must be carefully considered. However, the fact that compound **4** (phenyl group tethered to the exo-amine group at position 6 by a propyl linker) has the highest docking score for the A2A receptor, whereas compound **3** (corresponding molecule with a boron cluster, Figure 3) has the highest docking score for the A3 receptor could be of interest.

#### 2.3.2. Sugar Configuration

To determine the effects of the sugar stereochemistry on the ligand binding, β-d-ribofuranose containing compounds (**1**–**2** and **9**–**10**) were compared to β-d-arabinofuranose containing compounds (**5**–**6** and **7**–**8**, respectively), Figure 3, Table S1. It should be noted that the largest differences between the geometric scores and desolvation energies of the ribofuranose- and arabinofuranose-containing derivatives are observed when the phenyl ring is attached to the scaffold by a rigid ethynyl spacer (compounds **2** and **6**, Figure 5 and Figure 7). This result shows that the protein-ligand interactions are influenced not only by the presence of a phenyl ring but also possibly by the spacer flexibility. If the sugar moiety is arabinofuranose, functionalizing the ligand with a phenyl group in the 2 position via a rigid ethynyl linker might increase its affinity for the A2A receptor.

For the A3 receptor, the nature of the linker and functional group has a smaller effect on the influence of the sugar configuration (Figure 7 and Table S2). Therefore, this moiety could impact the ligand selectivity for the A3 receptor.

#### 2.3.3. Spacer Flexibility

**Figure 8:**
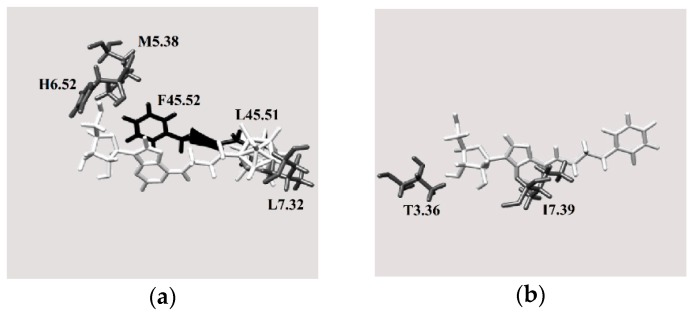
Views of the A2A receptor binding pocket showing the best docked poses of (**a**) compound **3** and (**b**) compound **4** (white: ligand, gray: transmembrane residues, black: extracellular amino acids).

The influence of spacer flexibility on A2A and A3 receptor-ligand interactions was already mentioned above. The geometric score profiles and desolvation energies reveal the significant effects of the spacer flexibility and length (score profiles shown in Figure 5 and Figure 8, energy profiles shown in Figure 6 and Figure S4). Indeed, the molecules with the highest geometric scores and lowest desolvation energies have flexible (**7**, **8**, **9**, **10**, **13** and **14**, Figure 5) and/or long spacers (**3**, **4**, **11** and **12**, Figure 5).

#### 2.3.4. Binding Pocket

Figure 8b shows the A2A binding pocket for the best docked pose of molecule **4** (Figure 3), which is best ligand for this receptor based on the in silico test results (Figure 5 and Figure 7). Based on the number of unfavorable interactions (i.e., the number of clash contacts, or atom pairs separated by a distance of less than the sum of their van der Waals radii, Figure S5), substituting the phenyl ring with a carborane cage is unfavorable, although the geometric score is essentially unchanged after this modification (cf. the results for compounds **3** and **4**). This result might be due to the fact that new interactions between the ligand and the extracellular L45.51 and F45.52 residues arise when the phenyl group is substituted by a boron cluster (Figure 8b) (the reference for the residue distribution was the sequence of human A2 AR, UniProtKB code: P29274). These amino acids probably force the boron cluster to lie in a pocket that is too small, resulting in unfavorable protein-ligand interactions. In contrast, molecule **4** has a more complementary size and shape to the A2A into this adenosine receptor.

**Figure 9:**
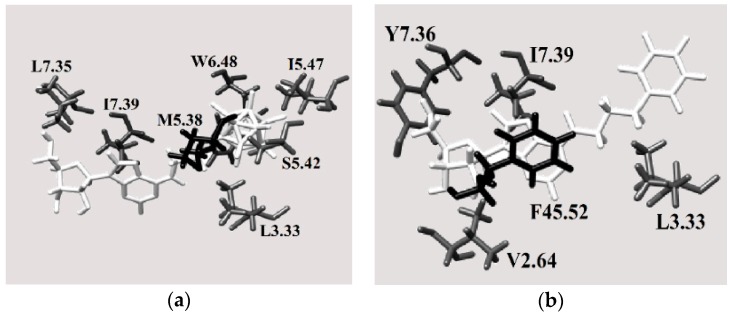
Views of the A3 receptor binding pocket for the best docked poses of (**a**) compound **3** and (**b**) compound **4** (white: ligand, gray: transmembrane residues, black: extracellular amino acids).

For the A3 receptor, replacing the phenyl ring with a boron cluster results in more favorable protein-ligand interactions (Figure 9). Indeed, of molecules **1**–**16**, molecule **3** was identified as the hit compound for A3 AR among the molecules containing carborane group. As shown in Figure 9b, when the phenyl group is substituted by a boron cluster to give molecule **3**, the ligand interacts with more transmembrane amino acids. Therefore, it was concluded that this region is fundamental for ligand binding to the A3 receptor. This hypothesis was also supported by the relative numbers of clash contacts for compounds **3** and **4** (Figure S6). Indeed, this parameter is reduced by 33% when the phenyl ring is replaced with a boron cluster, thus suggesting that, from a geometric shape complementarity point of view, boron cluster structure fits the A3 receptor channel in a better way if compared to phenyl group. A possible reason could be given by the reduced mobility of boron cluster which anchors the molecule more efficiently to inner surface of the channel. Anyway, the cause of this clash contacts reduction is unclear taking into account that the van der Waals volume of the carborane cage is ca. 50% higher than that of the phenyl group [38].

Nevertheless, the boron cluster in molecule **3** interacts with more intramembrane amino acids (L3.33, S5.42, I5.47 and W6.48) (the reference for the residue distribution was the sequence of human A3 AR, UniProtKB code: P0DMS8). These interactions are more favorable than those between the phenyl ring in molecule **4** and the surrounding protein residues (Figure 9b). Interestingly, when the phenyl ring is substituted by a boron cluster, the molecule can penetrate further into the receptor binding pocket towards the intramembrane region. Indeed, molecule **3** interacts with only one extracellular residue, which is located near the intramembrane region of the protein, whereas molecule **4** interacts with one residue located deeper in the extracellular region.

Overall, these results indicated that the distal region of extracellular loops of both the A2A and A3 receptors (blue in Figure 1) hinders ligand binding to them, whereas interactions between the ligand and the protein at extra/intramembrane interface, where binding pocket is located, are favorable. Indeed, the binding pockets of the best ligands (Figure 8b and Figure 9b) are mostly localized in the transmembrane region, and these ligands exhibit the fewest clash contacts with the protein residues in rigid docking (Figures S5 and S6). Thus, the distal extracellular residues in A2A and A3 might not interact unfavorably with ligands; the A2A and A3 binding sites for ligands not fitting sterically (e.g., compound **11**) involve more distinct amino acids at the extracellular loop and have more clash contacts than those of the best ligands (Tables S1 and S3 for A2A and Tables S2 and S4 for A3). It is well-founded that extracellular domains of G protein-coupled receptors can be crucial for ligand binding and for activation/inhibition of the adenosine receptors. It would be of interest therefore to analyze the molecules described herein also against other members of this group of receptors to acquire information about their specificity for different members of this protein family. These study are however beyond the scope of the present communication.

### 2.4. Synthesis of Compounds ***3***,***4*** and ***11***,***12***, Which Contain a Boron Cluster or Phenyl Group

**Figure 10:**
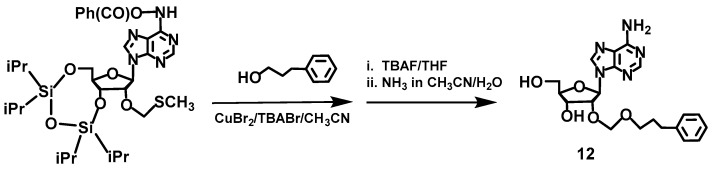
Synthesis of 2′-*O*-(3-phenylpropyleneoxymethyl]-adenosine (**12**) from a key intermediate 6-*N*-benzoyl-3′,5′-*O*,*O*-(tetraisopropyldisiloxane-1,3-diyl)-2′-*O*-methylenethiomethyladenosine.

Compounds **3** [19], **4** [50] and **11** [51] were synthesized as described. Compound **12** was analogously obtained as **11** using 3-phenyl-1-propanol instead of 3-(1,12-dicarba-*closo* dodecaboran-1-yl)-1-propanol (Figure 10). Thus, first, 6-*N*-benzoyl-3′,5′-*O*,*O*-(tetraisopropyldisiloxane-1,3-diyl)adenosine was prepared from adenosine according to the a previously reported procedure [52]; then, a reaction with DMSO in a mixture of acetic acid/acetic anhydride [53] provided a key intermediate 6-*N*-/benzoyl-3′,5′-*O*,*O*-(tetraisopropyldisiloxane-1,3-diyl)-2′-*O*-methylenethiomethyl-adenosine. The treatment of the intermediate with 3-phenyl-1-propanol and subsequent removal of the protecting groups produced compound **12**.

### 2.5. Radioligand Assay

The radioligand replacement assay based on the competition binding of the tested compound and a ligand with known affinity toward the receptor was performed under contractual service agreement with Plataforma de Screening de Farmacos (USEF), 15782 Santiago de Compostela, Spain. As the radioligand for the A2A receptor [^3^H]-ZM241385, a 2,8-substituted[1,2,4]triazolo[1,5-*a*][1,3,5]triazine, an adenine isoster and a high-affinity antagonist, which is selective for the adenosine A2A receptor, was used. As the standard control for the studied receptor-binding CGS15943, non-nucleoside agonist was applied. Non-specific binding was determined in the presence of NECA.

For the A3 receptor 10 nM [^3^H]-NECA, a 5′-*N*-ethylcarboxamide adenosine derivative, was used. Non-specific binding was determined in the presence of a high concentration of R-PIA, a N^6^-(2-phenylisopropyl)adenosine, a specific agonist for A1 receptor. The binding affinities were measured as a percent of the radioligand displacement by the tested compounds and are shown in Table 1. For both pairs of compounds, the binding of the adenosine ligands that were modified with the phenyl group is more efficient than that of the counterparts modified with the boron cluster. The high, in nM range, binding affinity of phenyl modified compound **4** (K_i_ = 7.5 nM) should be pointed out as a good starting point for the further improvements [54,55].

**Figure 11:**
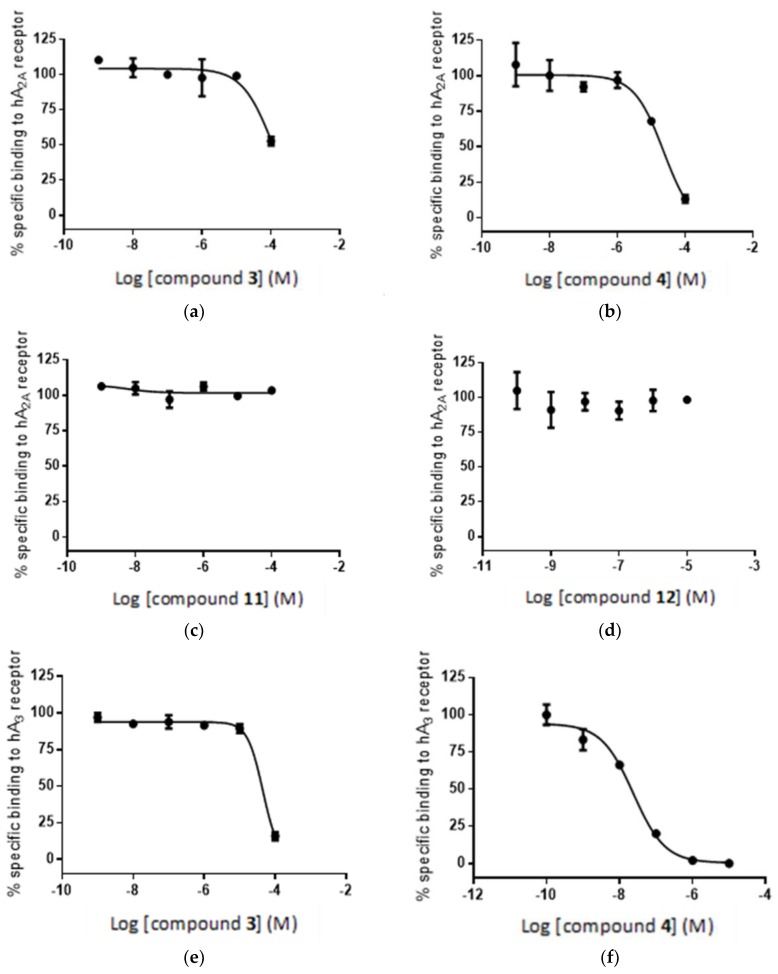

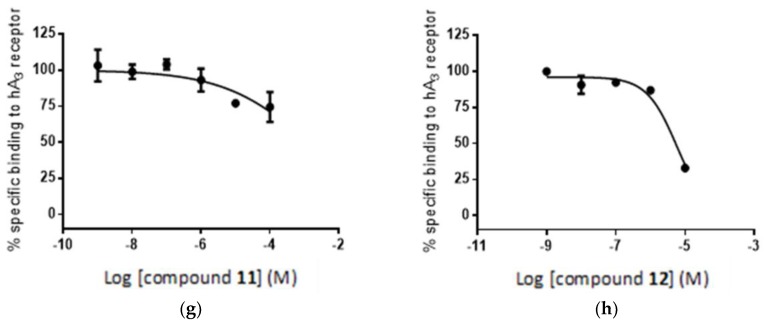
Radioligand competition binding assay: specific binding of compounds **3**, **4**, **11** and **12** to the adenosine receptors A2A (**a**–**d**) and A3 (**e**–**h**) (see Experimental Section).

Interestingly the concentration-response curves of the compounds are qualitatively, if not quantitatively, consistent with the results of the in silico study. Thus, Figure 11a,b shows a high concentration-dependent binding of compounds **3** and **4** to the A2A receptor, where **4** > **3**, which is qualitatively consistent with the in silico calculation. In the case of compounds **11** and **12**, a substantial decrease in geometric score is observed for both compounds (Figure 5), which is reflected in the flat, non-binding, concentration-response curves (Figure 11c,d). However, the differences in binding affinities are much smaller (Figure 11c,d) than the expected values based on the geometric score disparity (Figure 5).

**Figure 12:**
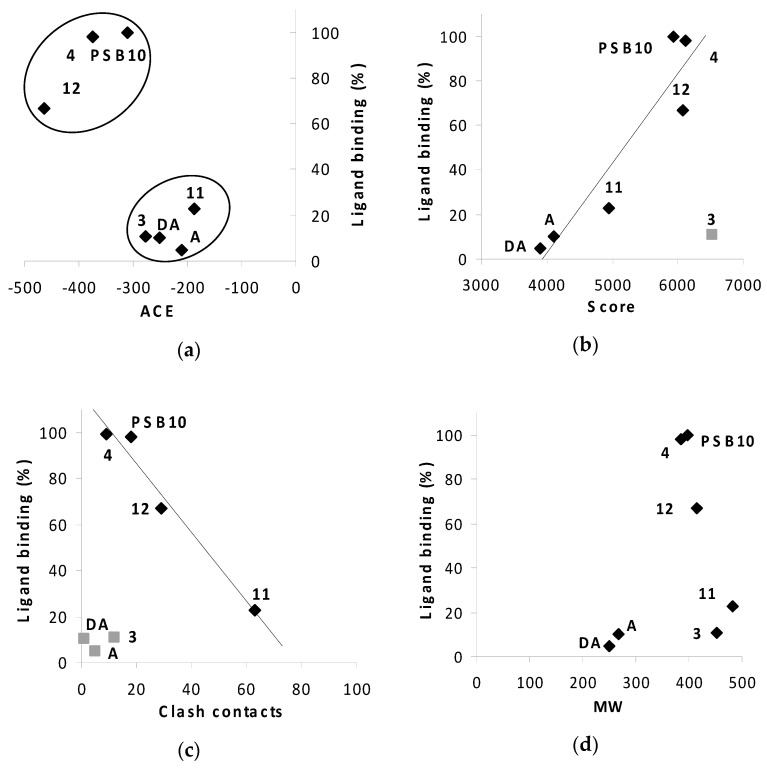
Relationships of desolvation energy, ACE (**a**), geometric scores (**b**) and clash contacts (**c**), calculated in silico, and ligand binding in vitro for A3 receptor, for the compounds **3**, **4**, **11**, **12**, PSB 10, adenosine (A) and 2′-deoxyadenosine (DA) as ligands; The relationship of ligand binding and molecular weight (MW) (**d**). Ligand binding was determined in radioligand competition binding assay as described in Materials and Methods, and expressed as % inhibition of a specific binding at 10 µM. Values of MW [D] for **3**, **4**, **11**, **12**, PSB 10, adenosine and 2′-deoxyadenosine are 451.53, 385.42, 481.56, 415.44, 398.67, 267.24 and 251.24, respectively.

For the binding of compounds **3**, **4** and **11**, **12** to receptor A3, a similar but less consistent relationship between in silico calculations and biological screening can be observed. Again, compounds **3** and **4** show reasonable binding to the receptor (Figure 11d,e) as predicted in the in silico calculations (Figure 7), although the order of affinities is reversed: **3** > **4** for the in silico assay and **4** > **3** in the biological test. In other words, the radioligand assay showed lower affinity of compound **3** (boron cluster modification) to A3 receptor than that of compound **4** (phenyl group modification), though the geometric scores (6530 vs. 6120) and clash contacts (11 vs. 18) do not reflect the respective differences in compounds **3** and **4** affinity for the receptor (Figure 7 and Table S2). Consequently, the observed difference in binding affinity between compounds **3** and **4**, in the radioligand replacement assay (Table 1, Figure 11d,e) is much higher than the predicted value from the geometric scores (Figure 7). For the compound pair of **11** and **12** (Figure 11f,g) the relationship goes back to the previous correlation in both the in silico assay and the biological test: the phenyl modification corresponds to higher affinity. These in vitro results are consistent with the observed trend in in silico study (Figure 12).

The observed consistency in trends of the effect of the boron cluster or phenyl modification on the adenosine binding to the A2 and A3 receptors for both in silico and wet assays, although only approximate, is important as a base for further improvements.

In silico and in vitro results for A3 receptor and compounds **3**, **4**, **11**, **12** and adenosine, or 2′-deoxyadenosine show similar trends (Figure 12a–c). The higher the score, the better the ligand binding to A3 receptor for **4**, **11**, **12**, PSB 10, adenosine and 2′-deoxyadenosine (Figure 12b, correlation R^2^ = 0.884). The score above 5000–5200 characterizes active compounds with an exception of compound **3** (outlier, Figure 12b). The reason for this discrepancy needs furthers study. The ACE parameter distinguish PSB 10 (highly specific ligand) and phenyl-modified compounds, **4** and **12**, with low ACE value and high activity form lower active adenosine or 2′-deoxyadenosine (nonspecific ligands) and boron cluster-modified compounds, **3** and **11** (Figure 12b). Notably, the clash contacts *versus* radioligand inhibition relationship was also convergent for almost all compounds compared (Figure 12c). The higher the clash contacts value, the worse the ligand binding to A3 receptor of **4**, **11**, **12** and PSB 10 (Figure 12c, correlation R^2^ = 0.96). Based on the correlation data we suggest that ACE can be the most specific parameter differentiating the compounds activity. We notice, that compounds with ACE <−300 were active to the receptor in in vitro study. Moreover, we can presume that compounds with activity equal to or greater than 40% (at 10 µM) can exhibit specific interactions with A3R. The theoretical and experimental properties of compounds **4** and **12** are in close proximity to selective A3R ligand, PSB 10 in all analyses (Figure 12a–c). On the other hand, with reference to low number of clash contacts and concomitant low binding to A3R, the compound **3** is similar to non-selective adenosine or 2′-deoxyadenosine. In light of these observations, compound **11** with low score, high number of clash contacts and the highest molecular weight (whatever it means for the adenosine receptor), appears to be the worst candidate for the A3 receptor.

The plot analysis of the ligand binding *versus* molecular weight (MW) of compounds did not show any clear relationships (Figure 12d). Additionally, ligand efficiency instead of ligand binding plotted against in silico parameters (Figure 7S, Supplementary Information) yielded similar results like for ligand binding factor (Figure 12a–c).

The consistency between in silico and wet assay results is still a difficult challenge. There are several possible reasons for not perfect overlapping of the calculation and experimental results described herein. First, despite the use of a new in boron cluster field calculation protocol based on the PatchDock server, the effects of the unique boron cluster properties on the action of molecules that target the A2A and A3 adenosine receptors could not be included into calculations. Second, for the docking experiments, the A2A receptor based on the structure that bound the endogenous adenosine, which is an agonist and fixes the receptor in the active conformation, was selected. Though, based on their structural similarity to adenosine, compounds **1**–**16** are likely agonists too, their agonist properties are currently not proven. Furthermore, the structure of the receptor A3 was not based on the crystal structure (as in the case of A2A), which is not yet available but is based on the homology modeling which introduces additional uncertainty. For receptor A3, the agonist radioligand was consistently used. Finally, the access to commercial radioligands with specific agonist or antagonist properties is limited. Thus, fitting the AR receptor structure, which is fixed in the agonist or antagonist conformation, with a tested ligand with agonist or antagonist properties, which are often unknown, in the docking experiment is difficult. Moreover, for the best result comparison of the in silico study and radioligand replacement assay, the radioligand with the identical agonist or antagonist properties to that in the docking experiment should be used in the biological test. Lack of conformity of all factors may affect the accuracy of prediction of the ligand behaviour in the biological environment based on the docking experiments. Therefore it may be of interest that compound **3** tested for affinity to A2A receptor using [^3^H]-ZM241385 antagonist radioligand described herein, displayed only moderate affinity, but it showed considerably higher inhibitory property when [^3^H]-CGS 21680, an agonist radioligand was used [18]. The reasonable, albeit not perfect overlapping of in silico and wet assay results described herein, while based on small set of compounds is encouraging and prompt study of larger library of adenosine derivatives using reported methodology.

## 3. Materials and Methods

### 3.1. General Information

Commercially available chemicals were of reagent grade and used as received. Adenosine and 2’-*O*-deoxyadenosine were purchased from Pharma-Waldhof GmbH (Düsseldorf, Germany), 3-phenylpropane-1-ol was from Sigma-Aldrich (Steinheim, Germany). Solvents were purchased in the highest available quality. Column chromatography was performed on silica gel 230-400 mesh and TLC was performed on silica gel F254 plates, both purchased from Sigma-Aldrich (Steinheim, Germany). 

^1^H-NMR spectra were recorded on a Bruker Avance III 600 MHz spectrometer. The spectra for ^1^H nuclei were recorded at 600.26 MHz using a deuterated solvent as a standard All chemical shifts are reported in ppm relative to the internal standards. UV measurements were performed with a GBC Cintra10e UV-VIS spectrometer (Dandenong, Australia). Samples for UV experiments, ca. 0.5 A_260_ ODU for each compound, were dissolved in 96% C_2_H_5_OH or CH_3_OH. The measurement was performed at ambient temperature. 

### 3.2. Synthesis of 2′-O-(3-Phenylpropyleneoxymethyl)-adenosine (***12***)

6-*n*-Benzoyl-3′,5′-*O*,*O*-(tetraisopropyldisiloxane-1,3-diyl)-2′-*O*-methylenethiomethyladenosine (1 eq, 40 mg, 0.059 mmol) was dissolved in acetonitrile (0.5 mL) then was treated with 3-phenylpropane-1-ol (2.7 eq., 22 mg, 0.16 mmol) at the presence of copper(II) bromide (1.1 eq, 14 mg, 0.065 mmol) and tetrabutylammonium bromide (1.1 eq, 21 mg, 0.065 mmol) as activators. The reaction progress was monitored by TLC (CH_2_Cl_2_/CH_3_OH, 9:1). After reaction completion (ca. 48 at room temperature) solvent was evaporated under reduced pressure, then the oily residue was dissolved in dichloromethane (2 mL). The resultant solution was washed with water (3 × 1 mL) then the organic fraction was dried over anhydrous magnesium sulfate and evaporated to dryness under vacuum. Next, the crude product without purification was dissolved in THF (1.2 mL) then TBAF (1 mL, 3.5 mmol) was added. After 15 min to the reaction mixture a pyridine/methyl alcohol/water (3:1:1, 2.5 mL) followed by ion exchange resin Dowex 50Wx8 in pyridinium form, was added. After 30 min the ion exchange resin was filtered off and washed with pyridine/methyl alcohol/water (3:1:1, 3 × 5 mL). The filtrate and washings were combined together then whole was evaporated to dryness under vacuum yielding crude product. The crude product was purified by silica gel column chromatography (5 g, 230–400 mesh) using a linear gradient of CH_3_OH in CH_2_Cl_2_ (0–5%) as a eluting solvent system. The obtained 6-*N*-benzoyl-2′-*O*-(3-phenylpropyleneoxymethyl)adenosine (ca. 30 mg) was dissolved in CH_3_CN (0.2 mL) then concentrated aq. ammonia solution was added (2 M, 2 mL). After 2 h at room temperature (TLC control, CH_2_Cl_2_/CH_3_OH, 9:1) solvents were evaporated under vacuum then final product was purified by silica gel column chromatography (5 g, 230–400 mesh) using a linear gradient of CH_3_OH in CH_2_Cl_2_ (0–10%) as a eluting solvent system. Yield: 9 mg. TLC (CH_2_Cl_2_/CH_3_OH, 9:1 *v*/*v*): R_f_ = 0.64; UV-Vis (95% C_2_H_5_OH) λ, nm: 249 (min), 280 (max); ^1^H-NMR (600 MHz, CDCl_3_) δ (ppm): 2.34–2.37 (m, 2H, CH_2_), 3.33–3.35 (m, 2H, CH_2_), 3.81–3.95 (m, 2H, H-5′, 5″), 4.075 (d, 2H, CH_2_-O, *J* = 6.6 Hz), 4.225 (d, 1H, H-4′, *J =* 3.0 Hz), 4.51–4.52 (m, 1H, H-3′), 4.735 (d, 1H, H-2′, *J =* 6.6 Hz), 6.305 (d, 1H, H-1′, *J* = 6 Hz), 7.04–7.09 (m, 2H, phenyl ring), 7.18 (t, 2H, NH_2_, *J* = 7.8 Hz), 7.58 (t, 2H, phenyl ring, *J* = 7.8 Hz), 7.67–7.68 (m, 1H, phenyl ring), 8.065 (d, 1H, H-8, *J* = 1.8 Hz), 8.725 (d, 1H, H-2, *J* = 7.8 Hz).

### 3.3. Docking Analysis

Computational docking simulations were conducted using two different web services: SwissDock (http://www.swissdock.ch) and PatchDock v1.3 (http://bioinfo3d.cs.tau.ac.il/PatchDock). The former web service is based on the EADock DSS (Evolutionary Algorithm for Docking) software [35]. Evolutionary algorithms are iterative stochastic optimization procedures in which an initial population of solutions is generated and evaluated with respect to a set of constraints described by the fitness function.

The PatchDock algorithm consists of three main phases [46]. In the first phase, the surface of the molecule is computed, and a segmentation process is subsequently performed to determine the geometric patches (concave, convex and flat surface sections). Then, only the patches with “hot spot” residues are retained and matched via a hybrid of the Geometric Hashing and Pose-Clustering matching techniques. During this step, the concave patches are matched to convex ones, and flat patches are matched with any type of patches. The resulting candidate complexes are examined to discard all complexes with unacceptable overlap between the receptor and ligand atoms. Finally, the remaining candidates are ranked based on their geometric shape complementarity scores. For the preliminary A2A receptor docking studies, the input target consisted of the A2A structure extracted from the A2A protein/adenosine complex X-ray structure (PDB code: 2YDO) [28]. All the molecules in Figure 3 and Figure 4 were considered to be ligands, and their input files for the SwissDock server were generated with Chem3DPro 16.0 and then converted into the “.mol2” format with UCSF Chimera [29]. For the PatchDock simulations, all the compounds were drawn in Chem3DPro 16.0 and saved as “.pdb” files. The docking studies were performed using a clustering RMSD (root mean square deviation, parameter used to discard redundant solutions) of 4.0 Å. All the simulations were conducted without specifying a region of interest (ROI) to ensure that the chosen docking methods could locate the correct binding pocket.

### 3.4. Radioligand Replacement Assay for Human Adenosine Receptors

The 10 mM stock solutions were prepared by dissolving 0.5–1 mg of the tested compounds in suitable volume of DMSO. Next, dilutions in binding buffer were prepared to obtain concentration ranges 10^−10^–10^−5^ or 10^−9^–10^−4^ depending upon the compound potency. The concentration-response curves of the standard controls were made. For this purpose A3R antagonist, MRS 1220, and A2AR antagonist, CGS 15943 were used for the binding study in the same buffer/vehicle system as for tested compounds.

Adenosine A2A receptor competition binding experiments were carried out in a multiscreen GF/C 96-well plate (Millipore, Madrid, Spain) pretreated with binding buffer (Tris-HCl 50 mM, EDTA 1 mM, MgCl_2_ 10 mM, 2 U/mL adenosine deaminase, pH = 7.4). In each well was incubated 5 μg of membranes from Hela-A2A cell line (Lot: A001/18-10-2010, protein concentration = 4288 μg/mL), 3 nM [3H]-ZM241385 (50 Ci/mmol, 1 mCi/mL, ARC-ITISA 0884) and compounds studied and standard. Non-specific binding was determined in the presence of NECA 50 μM (Sigma E2387, St. Louis, MO, USA). The reaction mixture (Vt: 200 μL/well) was incubated at 25 °C for 30 min, after was filtered and washed four times with 250 μl wash buffer (Tris-HCl 50 mM, EDTA 1 mM, MgCl_2_ 10 mM, pH = 7.4), before measuring in a microplate beta scintillation counter (Microbeta Trilux, PerkinElmer, Madrid, Spain).

Adenosine A3 receptor competition binding experiments were carried out in a multiscreen GF/B 96-well plate (Millipore, Madrid, Spain) pretreated with binding buffer (Tris-HCl 50 mM, EDTA 1 mM, MgCl_2_ 5 mM, 2 U/mL adenosine deaminase, pH = 7.4). In each well was incubated 70 μg of membranes from Hela-A3 cell line (Lot: A003/13-04-2016, protein concentration = 2449 μg/mL), 10 nM [3H]-NECA (27.6 Ci/mmol, 1 mCi/mL, Perkin Elmer NET811250UC) and compounds studied and standard. Non-specific binding was determined in the presence of R-PIA 100 μM (Sigma P4532, St. Louis, MO, USA). The reaction mixture (Vt: 200 μL/well) was incubated at 25 °C for 180 min, after was filtered and washed six times with 250 μL wash buffer (Tris-HCl 50 mM pH = 7.4), before measuring in a microplate beta scintillation counter (Microbeta Trilux, PerkinElmer, Madrid, Spain).

## 4. Conclusions

This work describes a practical protocol for evaluating adenosine derivatives as potential ARs ligands in silico. In this approach, the structural features of the ligand and their effect on interactions with receptor proteins can be assessed. Here, the binding of adenosine ligands modified with phenyl groups was compared to that of ligands modified with inorganic modifier, a boron cluster. The results can be used to guide future biological tests and identify lead molecules within this class of compounds for follow-up studies. In this work, compounds **3** and **4** (one with a boron cluster and one with a phenyl group) were found to be the most promising ligands. However, the observation made that a boron cluster can hinder the ligand from entering the receptor binding pocket calls for caution in the cases where the interaction between a cluster and spatially limited protein cavity can be expected. It shows also that the 50% higher van der Waals volume of the carborane cage than that of the rotating phenyl group can make a difference. This observation can be of importance not only for adenosine ligands/AR system but for medicinal chemistry of boron cluster in general. On the other hand, it should be stressed that the performed in silico screening was based on the effect of this moiety on the rigid docking and the space fitting approach, and that other unique, potentially beneficial, properties of boron clusters were not included due to the modeling softwares limitations.

In silico as well as in biological assay results show the noteworthy compatibility in trends although not quantitative consistency. Current our work is focused on improvements of the described modeling methodology to make it better applicable to A3 and other adenosine receptors. Further works both in silico and experimentally are also ongoing to provide more insight into adenosine modifications for more selective binding to different adenosine receptors and wet assays to validate this in silico approach.

## Figures and Tables

**Table 1:** Specific binding of the compounds **3**, **4**, and **11, 12** to adenosine receptors A2A and A3 in radioligand competition binding assay.

Compound	% Inhib. 10 μM A2A	% Inhib. 10 μM A3	K_i_ (nM) A3
Adenosine 2′-deoxyadenosine **3** **4** **11** **12**	2 ± 1	10 ± 1	n.d.
	3 ± 1	5 ± 1	n.d.
	2 ± 2	11 ± 4	n.d.
	32 ± 1	98 ± 1	7.5
	1 ± 1	23 ± 3	n.d.
	2 ± 1	67 ± 1	2208

n.d. = not determined.

## Supplementary Materials

The following are available online, Figure S1, (a) Best docking pose of adenosine obtained with SwissDock web server and considering human A2A receptor (PDB code: 2YDO) with thioglucoside, crystallization helper molecules and without (b); Figure S2, (a) X-ray structure thermostabilized human A2A receptor with adenosine bound (PDB code: 2YDO), (b) PatchDock best-docked pose obtained with adenosine as a ligand and considering human A2A receptor (PDB code: 2YDO); Figure S3, (a) Best docking pose of adenosine obtained with PatchDock web server and considering human A2A receptor (PDB code: 2YDO), with crystallization helper molecule and without (b); Figure S4, Desolvation energy associated to the A3 protein-ligand interaction. ● for reference molecules (CF101, CF102, adenosine, 2′-deoxyadenosine, and NECA); ▲ for molecules bearing boron clusters (**1**, **3**, **5**, **7**, **9**, **11**, **13**, **15**); ◆ for ligands with phenyl ring (**2**, **4**, **6**, **8**, **10**, **12**, **14**, **16**); Figure S5, Clash contact involved in A2A-ligand interaction ● for reference molecules (regadenoson, apadenoson, CGS 21680, adenosine, 2′-deoxyadenosine and NECA); ▲ for molecules bearing boron clusters (**1**, **3**, **5**, **7**, **9**, **11**, **13**, **15**); ◆ for ligands with phenyl ring (**2**, **4**, **6**, **8**, **10**, **12**, **14**, **16**); Figure S6, Clash contact involved in A3-ligand interaction ● for reference molecules (CF101, CF102, adenosine, 2′-deoxyadenosine, and NECA); ▲ for molecules bearing boron clusters (**1**, **3**, **5**, **7**, **9**, **11**, **13**, **15**); ◆ for ligands with phenyl ring (**2**, **4**, **6**, **8**, **10**, **12**, **14**, **16**); Figure S7, Relationships of desolvation energy, ACE (a), geometric scores (b) and clash contacts (c) *versus* ligand efficiency for A3 receptor and for the compounds **3**, **4**, **11**, **12**, PSB 10, adenosine and 2′-deoxyadenosine as ligands. Table S1, Docking results of molecules **1**–**16** towards adenosine receptor A2A; Table S2, Docking results of molecules **1**–**16** towards adenosine receptor A3; Table S3, Binding pockets of molecule **1**–**16** for adenosine receptor A2; Table S4, Binding pockets of molecule **1**–**16** for adenosine receptor A3.
