# Supplementary material for: Comparative Study of Carborane- and Phenyl-Modified Adenosine Derivatives as Ligands for the A2A and A3 Adenosine Receptors Based on a Rigid in Silico Docking and Radioligand Replacement Assay
Source: Molecules. 2018 Jul 25;23(8):1846. doi: 10.3390/molecules23081846 (PMC6222516; doi:10.3390/molecules23081846)
Supplement: Supplementary file 1 [file molecules-23-01846-s001.pdf]

SUPPLEMENTARY INFORMATION

Comparative study of carborane- and phenyl-modified adenosine derivatives as ligands for the A2A and A3 adenosine receptors based on a rigid *in silico* docking and radioligand replacement assay

Marian Vincenzi,[1#] Katarzyna Bednarska[2#] and Zbigniew J. Leśnikowski[1*]

*[1]Laboratory of Molecular Virology and Biological Chemistry, Institute of Medical Biology of the Polish Academy of Sciences, 106 Lodowa St., 92-232 Lodz, Poland; [2]Laboratory of Experimental Immunology, Institute of Medical Biology, Polish Academy of Sciences, 106 Lodowa St., 93-232 Lodz, Poland; *correspondence: zlesnikowski@cbm.pan.pl; Tel.: +48-42-272-3629; #contributed equally to this work.*

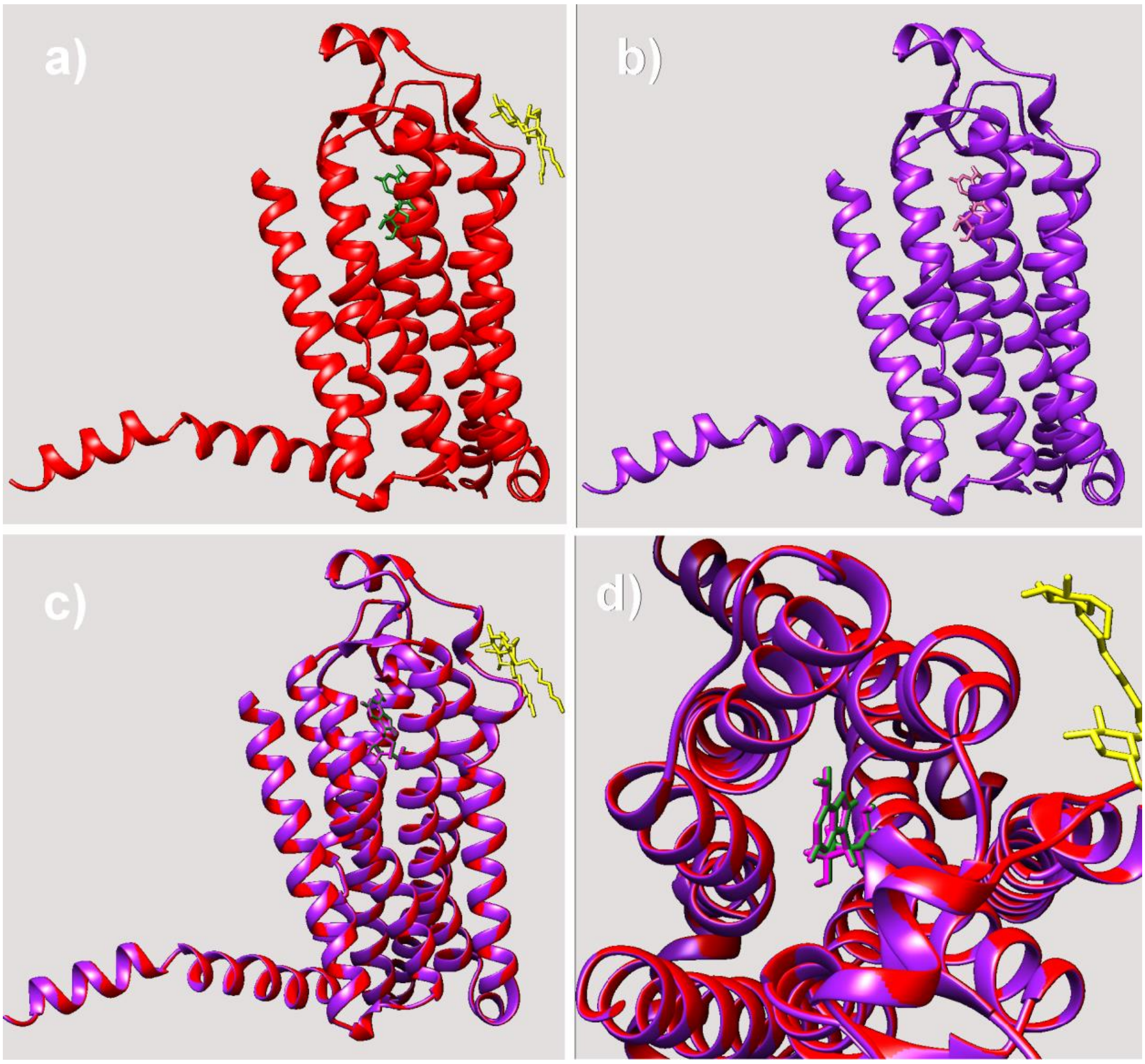

Figure S1. a) Best docking pose of adenosine obtained with SwissDock web server and considering human A2A receptor (PDB code: 2YDO), as protein input, with thioglucoside, crystallization helper molecules; b) SwissDock best-docked pose of adenosine considering human A2A receptor (PDB code: 2YDO), as protein input, without the crystallization helper molecules; c) superimposition of SwissDock best-docked poses obtained with and without the crystallization helper molecules. d) Enlargement of the superimposition viewed from the extracellular region, green for adenosine in the best-docked pose with thioglucoside, red for the protein in the best-docked structure with crystallization helper molecules, pink for adenosine in the best-docked pose without crystallization helper molecules, purple best-docked structure without crystallization helper molecules; yellow for crystallization helper molecules.

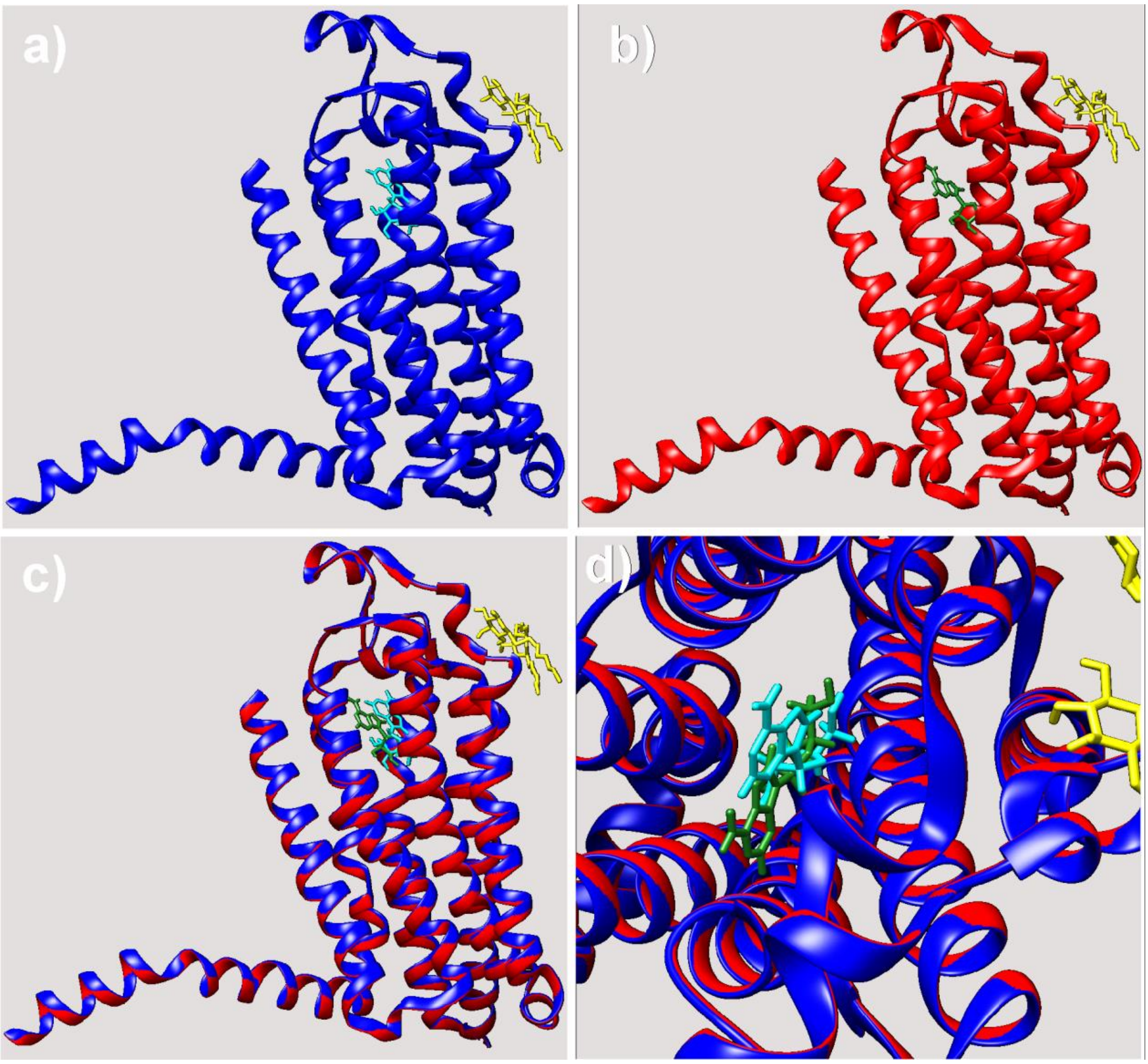

Figure S2. a) X-ray structure thermostabilized human A2A receptor with adenosine bound (PDB code: 2YDO),[28] b) PatchDock best-docked pose obtained with adenosine as a ligand and considering human A2A receptor (PDB code: 2YDO), as protein input, with the crystallization helper molecules. c) Superimposition of the reference complex (PDB code: 2YDO) and best docking pose of adenosine. d) Enlargement of the binding pockets viewed from the extracellular region; blue for receptor in the X-ray reference structure (PDB: 2YDO); cyan for adenosine in A2A receptor reference structure (PDB: 2YDO); red for the protein in the best-docked structure; green for adenosine in the best-docked pose; yellow for crystallization helper molecules.

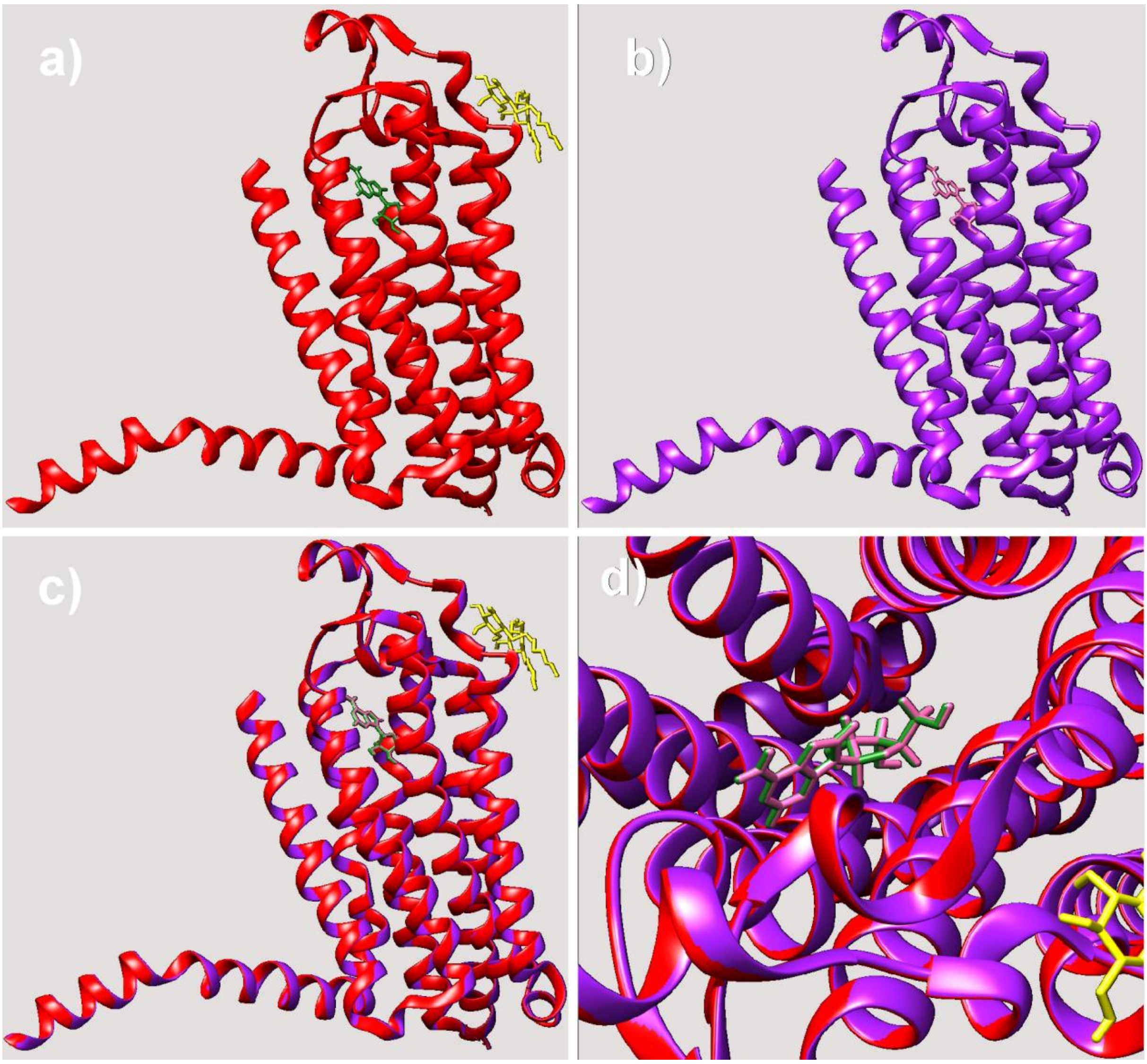

Figure S3. a) Best docking pose of adenosine obtained with PatchDock web server and considering human A2A receptor (PDB code: 2YDO), as protein input, with crystallization helper molecules, b) PatchDock best-docked pose of adenosine considering human A2A receptor (PDB code: 2YDO), as protein input, without the crystallization helper molecules, c) superimposition of PatchDock best-docked poses obtained with and without the crystallization helper molecules. d) Enlargement of the superimposition viewed from the extracellular region. Green for adenosine in the best-docked pose with thioglucoside; red for the protein in the best-docked structure with crystallization helper molecules; pink for adenosine in the best-docked pose without crystallization helper molecules; purple best-docked structure without crystallization helper molecules; yellow for crystallization helper molecules.

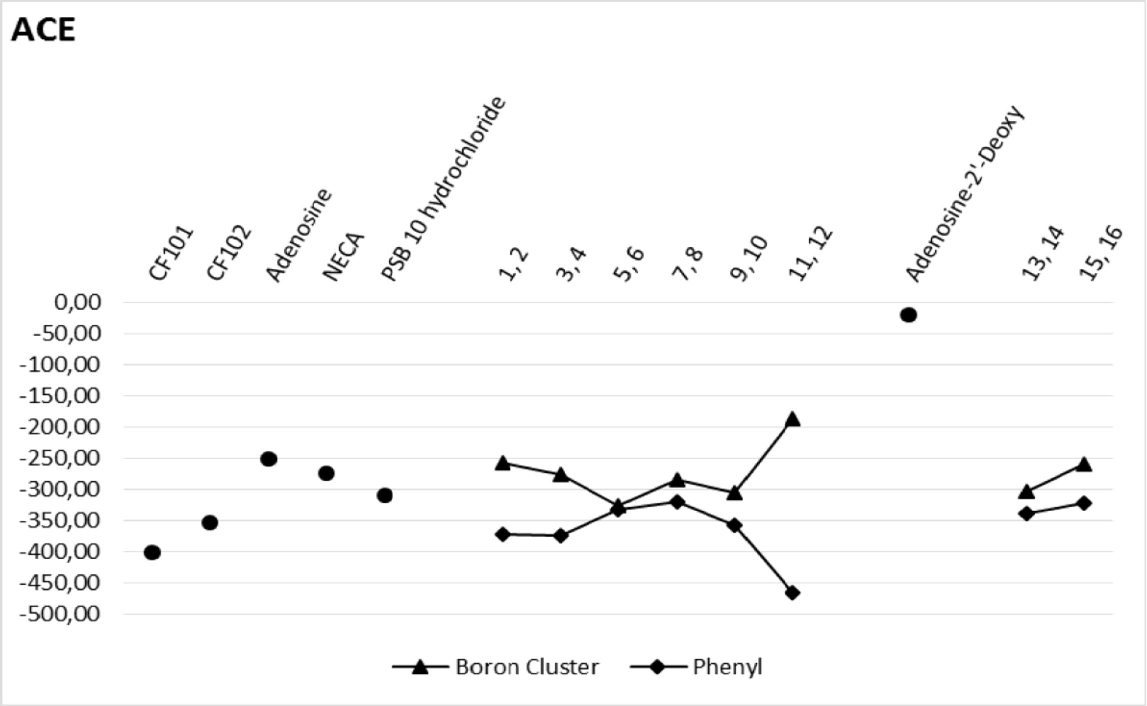

Figure S4. Desolvation energy (ACE) for the A3 protein-ligand interaction ● for reference molecules (CF101, CF102, adenosine, 2′-deoxyadenosine, NECA and PSB 10 hydrochloride); ▲ for molecules bearing boron clusters (**1**, **3**, **5**, **7**, **9**, **11**, **13**, **15**); ◆ for ligands with phenyl ring (**2**, **4**, **6**, **8**, **10**, **12**, **14**, **16**).

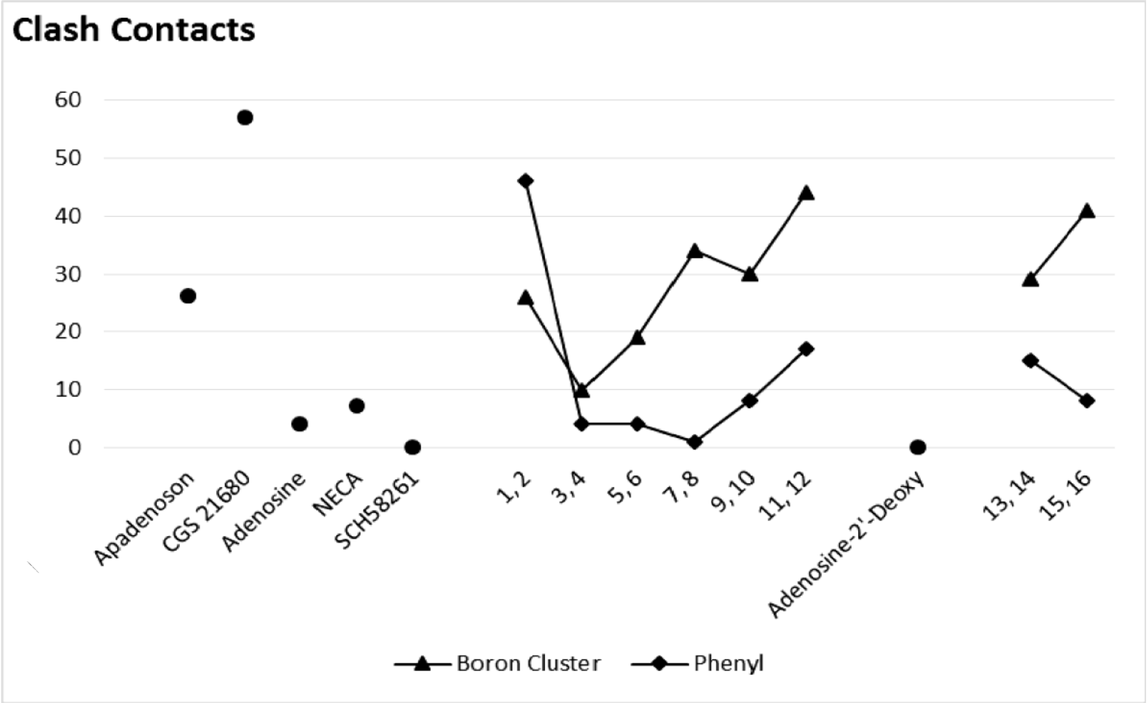

Figure S5. Clash contact involved in the A2A protein-ligand interaction ● for reference molecules (apadenoson, CGS 21680, adenosine, SCH58261, 2′-deoxyadenosine and NECA); ▲ for molecules bearing boron clusters (**1**, **3**, **5**, **7**, **9**, **11**, **13**, **15**); ◆ for ligands with phenyl ring (**2**, **4**, **6**, **8**, **10**, **12**, **14**, **16**).

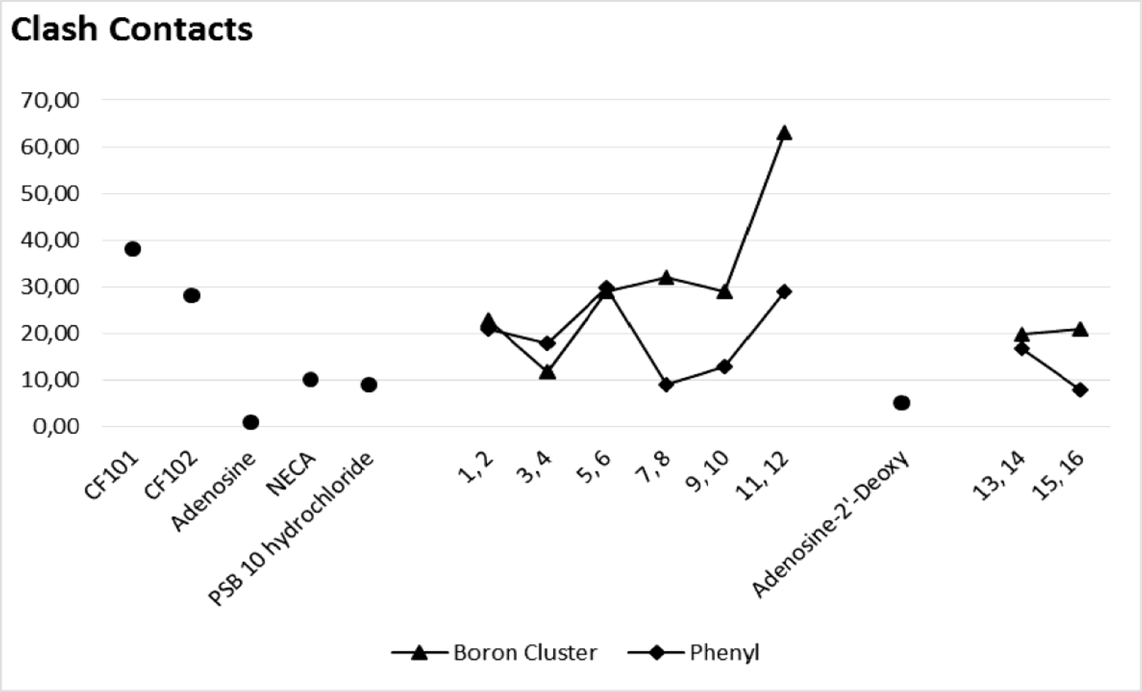

Figure S6. Clash contact involved in the A3 protein-ligand interaction ● for reference molecules (CF101, CF102, Adenosine, 2′-deoxyadenosine, NECA and PSB 10 hydrochloride); ▲ for molecules bearing boron clusters (**1**, **3**, **5**, **7**, **9**, **11**, **13**, **15**); ◆ for ligands with phenyl ring (**2**, **4**, **6**, **8**, **10**, **12**, **14**, **16**).

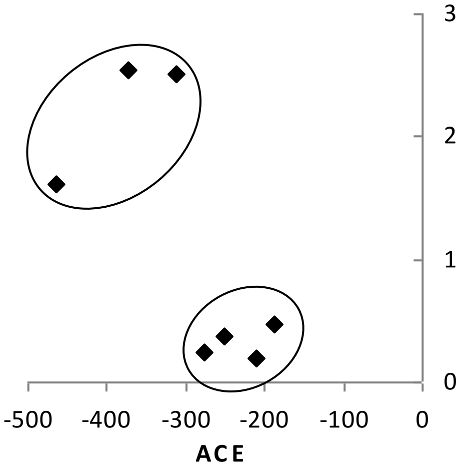

(a)

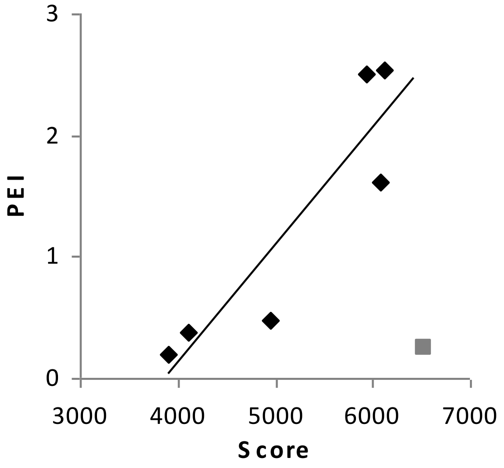

(b)

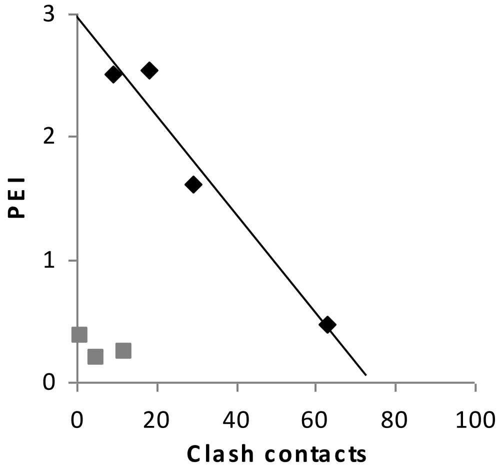

(c)

Figure 7S. Relationships of desolvation energy, ACE (a), geometric scores (b) and clash contacts (c) *versus* ligand efficiency for A3 receptor and for the compounds **3**, **4**, **11**, **12**, PSB 10, adenosine and 2′-deoxyadenosine as ligands. Ligand efficiency was expressed as PEI (potency efficiency index) calculated according to the equation PEI = (inhibition at 10 μM, ratio) / (MW, kD). PEI values of the compounds are 0.24, 2.54, 0.48, 1.61, 2.51, 0.37, 0.20, respectively.

Table S1. Docking results of molecule **1-16** towards adenosine receptor A2A.

Molecule	Score(1)	ACE(2)	Clash Contacts
**Apadenoson**	6606	-363.31	26
**CGS 21680**	6470	-334.18	3
**Adenosine**	4218	-207.74	4
**NECA**	4446	-216.00	7
**SCH58261**	5918	-284.37	0
**1**	5300	-263.44	26
**2**	4538	-44.88	46
**3**	6032	-272.99	10
**4**	6286	-281.97	4
**5**	5336	-273.61	19
**6**	5568	-299.64	4
**7**	5202	-277.51	34
**8**	6104	-297.73	1
**9**	5198	-245.86	30
**10**	6178	-297.22	8
**11**	4618	-220.47	44
**12**	5618	-224.77	17
**2′-Deoxyadenosine**	4266	-198.57	0
**13**	5000	-244.14	41
**14**	5216	-311.93	8
**15**	5108	-255.24	29
**16**	5106	-263.44	15

Score(1) - geometric shape complementarity score; ACE(2) (atomic contact energies) - desolvation free energies required to transfer atoms from water to a protein's interior.

Table S2. Docking results of molecule 1-16 towards adenosine receptor A3.

Molecule	Score[(1)]	ACE[(2)]	Clash Contacts
**CF101**	5922	-401.50	38
**CF102**	6344	-353.51	28
**Adenosine**	4110	-252.09	1
**NECA**	4686	-274.80	10
**PSB 10 hydrochloride**	5926	-310.94	9
**1**	5492	-256.90	23
**2**	5868	-372.38	21
**3**	6530	-276.46	12
**4**	6120	-373.31	18
**5**	5552	-326.72	29
**6**	6188	-331.94	10
**7**	5866	-283.52	32
**8**	6044	-320.62	9
**9**	5688	-305.78	29
**10**	6062	-356.64	13
**11**	4942	-186.65	63
**12**	6086	-465.12	29
**2′-Deoxyadenosine**	3898	-21.73	5
**13**	5314	-260.20	21
**14**	5452	-322.73	8
**15**	5518	-304.00	20
**16**	5580	-337.54	17

Score[(1)] - geometric shape complementarity score; ACE[(2)] (atomic contact energies) - desolvation free energies required to transfer atoms from water to a protein’s interior.

Table S3. Binding pockets of molecules **1-16** for adenosine receptor A2.

Molecule	Binding Pocket[(1)]		
	E[(2)]	T[(3)]	C[(4)]
**Apadenoson**	S2.65, T2.66, L45.51, F45.52, E169	L3.33, L7.32, I7.39, H7.43	
**CGS 21680**	S2.65, F45.52, E169, His264		
**Adenosine**		A2.61	
**NECA**	F45.52, E169	L7.32	
**SCH58261**		W6.48	
**1**	Ser77, F45.52, E169,	I2.64, V3.32, T3.36, W6.48, L6.51, H7.43	
**2**			K6.29, I8.47
**3**	L45.51, F45.52,	M5.38, H6.52, L7.32	
**4**		T3.36, I7.39	
**5**	F45.52, E169		
**6**	S2.65, F45.52, E169	A2.61, I2.64, M7.35, I7.39	
**7**	F45.52, E169	A2.61, W6.48, L6.51,	
**8**	S2.65	A2.61, M7.35, I7.39	
**9**	F45.52	A2.61, V3.32, T3.36, N5.42, C5.46, W6.48, L6.51, H6.52	
**10**	F45.52, E169	A2.61	
**11**	L45.51, F45.52	L7.32, Y7.36	
**12**	L45.51, F45.52, E169	I7.39	
**Adenosine-2′-Deoxy**	F45.52	A2.61, I7.39	
**13**		A2.61, V3.32, L3.33, T3.36, N5.42, W6.48, L6.51, H7.43	
**14**	F45.52	A2.61, I2.64, I7.39	
**15**	S2.65, F45.52, E169	A2.61, L3.33, T3.36, W6.48, L6.51	
**16**	F45.52	A2.61, V3.32, T3.36, L6.51, H6.52, M7.35	

Binding Pocket[(1)] - residues with more than 10 contacts; E[(2)] - extracellular region; T[(3)] - transmembrane region; C[(4)] - cytoplasmic region.

Table S4. Binding pockets of molecules **1-16** for adenosine receptor A3.

Molecule	Binding Pocket[(1)]		
	E[(2)]	T[(3)]	C[(4)]
**CF101**	M5.38	A2.61, V2.64, L3.32, L3.33, S5.42, F5.43, I5.47, L6.51	
**CF102**	F45.52	L3.32, W6.48, L7.35, I7.39	
**Adenosine**	F45.52	L3.32, L7.36, I7.39	
**NECA**	F45.52	L3.32, I7.39	
**PSB 10 hydrochloride**	F45.52	L3.32, L7.35, L7.36	
**1**		L6.51, N6.55, L7.35, L7.36, I7.39	
**2**	M5.38	L3.32, W6.48, L7.35, I7.39	
**3**	M5.38	L3.33, S5.42, I5.47, W6.48, L7.35, I7.39	
**4**	F45.52	V2.64, L3.33, L7.36, I7.39	
**5**	F45.52	A2.61, L3.32, L3.33, T3.36, S5.42, W6.48, L6.51	
**6**	F45.52	L6.51, L7.35, I7.39	
**7**	F45.52, M5.38	A2.61, L3.32, S5.42, I5.47, W6.48, L6.51, N6.55, I7.39	
**8**		W6.48, L6.51, L7.35, I7.39	
**9**	M5.38	A2.61, L3.32, L3.33, S5.42, W6.48, L6.51, I7.39	
**10**	F45.52,	L3.32, L7.35, I7.39	
**11**	L8, S2.65, Q45.51, F45.52, Q7.32	L7.36	
**12**		L3.33, S5.42, F5.43, I5.47, W6.48, S6.52	
**Adenosine-2′-Deoxy**			K6.32, K8.49
**13**	F45.52, M5.38	V2.64, L3.32, L6.51, N6.55, L7.36	
**14**	F45.52	L7.35, L7.36, I7.39	
**15**	F45.52	A2.61, L7.35, L7.36, I7.39	
**16**	F45.52	A2.61, V2.64, L6.51, L7.35, I7.39	

Binding Pocket[(1)] - residues with more than 10 contacts; E[(2)] - extracellular region; T[(3)] - transmembrane region; C[(4)] - cytoplasmic region.
